# Squamous cell carcinoma arising from chronic sacrococcygeal pilonidal disease: a case report

**DOI:** 10.1186/s12957-017-1129-0

**Published:** 2017-03-17

**Authors:** Nick Michalopoulos, Konstantinos Sapalidis, Styliani Laskou, Evangelia Triantafyllou, Georgia Raptou, Isaak Kesisoglou

**Affiliations:** 1 3rd Department of Surgery, Aristotle University of Thessaloniki, AHEPA University Hospital, St. Kyriakidi 1, 54636 Thessaloniki, Greece; 2 Pathology Department, Aristotle University of Thessaloniki, AHEPA University Hospital, St. Kyriakidi 1, 54636 Thessaloniki, Greece

**Keywords:** Pilonidal disease, Squamous cell carcinoma, Anal fistula

## Abstract

**Background:**

Sacrococcygeal pilonidal disease is a chronic, well-recognized entity, characterized by the recurrent formation of an abscess or draining sinus over the sacrococcygeal area. It is one of the most common surgical problems. Rarely, chronic inflammation and recurrent disease leads to malignant transformation, most commonly to squamous cell carcinoma (SCC).

**Case presentation:**

We describe an extremely unusual case of SCC developing in a 60-year-old patient with a chronic pilonidal sinus complicated by an anal fistula. After wide surgical excision of the pilonidal sinus and fistulas and because of the poor healing process 6 months later, colonoscopy and a percutaneous fistulography were performed, revealing an anal canal-pilonidal fistula. Patient was treated with a more radical surgical resection with a prophylactic loop colostomy, but healing was not accelerated. Multiple biopsies were then taken from the surgical site at the time, which revealed the development of SCC. CT and MRI imaging techniques revealed SCC partial invasion of the coccyx and sacrum. As a result, aggressive surgical approach was decided. Histological examination revealed moderately to poorly differentiated SCC, and the patient was treated with adjuvant radiation therapy postoperatively. Nine months later, recurrence was found in the sacrum and para-aorta lymph nodes and the patient died shortly after. We discuss the clinical features, pathogenesis, treatment options, and prognosis of this rare malignant transformation.

**Conclusions:**

The development of SCC in chronic pilonidal disease is a rare but serious complication. Symptoms are usually attributed to the sacrococcygeal pilonidal disease (SPD), and diagnosis is often made late by histological examination of biopsies. Malignant transformation should be suspected in chronic SPD with recurrent episodes of inflammation, repeated purulent discharge, poor healing, and chronic complex fistulas.

## Background

Pilonidal disease is a chronic disorder that primarily affects Caucasian boys and men between the ages of 15 and 40 years old, but rarely those over 50 years old [1–4]. It is characterized by the recurrent formation of an abscess or draining sinus over the sacrococcygeal or perianal area. It is a benign disease which, if left untreated, may result in multiple draining sinuses with chronic recurrent abscess, drainage, and soiling of clothing [5, 6]. Cases of necrotizing wound infections, sacral osteomyelitis, meningitis, and malignant degeneration associated with pilonodal disease have also been reported [7]. Malignant transformation occurs in about 0.1% of patients and usually involves squamous cell carcinoma (SCC) [2, 8, 9]. We report an extremely rare case of SCC developing in a 60-year-old man with chronic pilonidal disease complicated by an anal fistula. We also discuss the clinical features, pathogenesis, treatment options, and prognosis of this rare malignant transformation.

## Case presentation

**Fig. 1:**
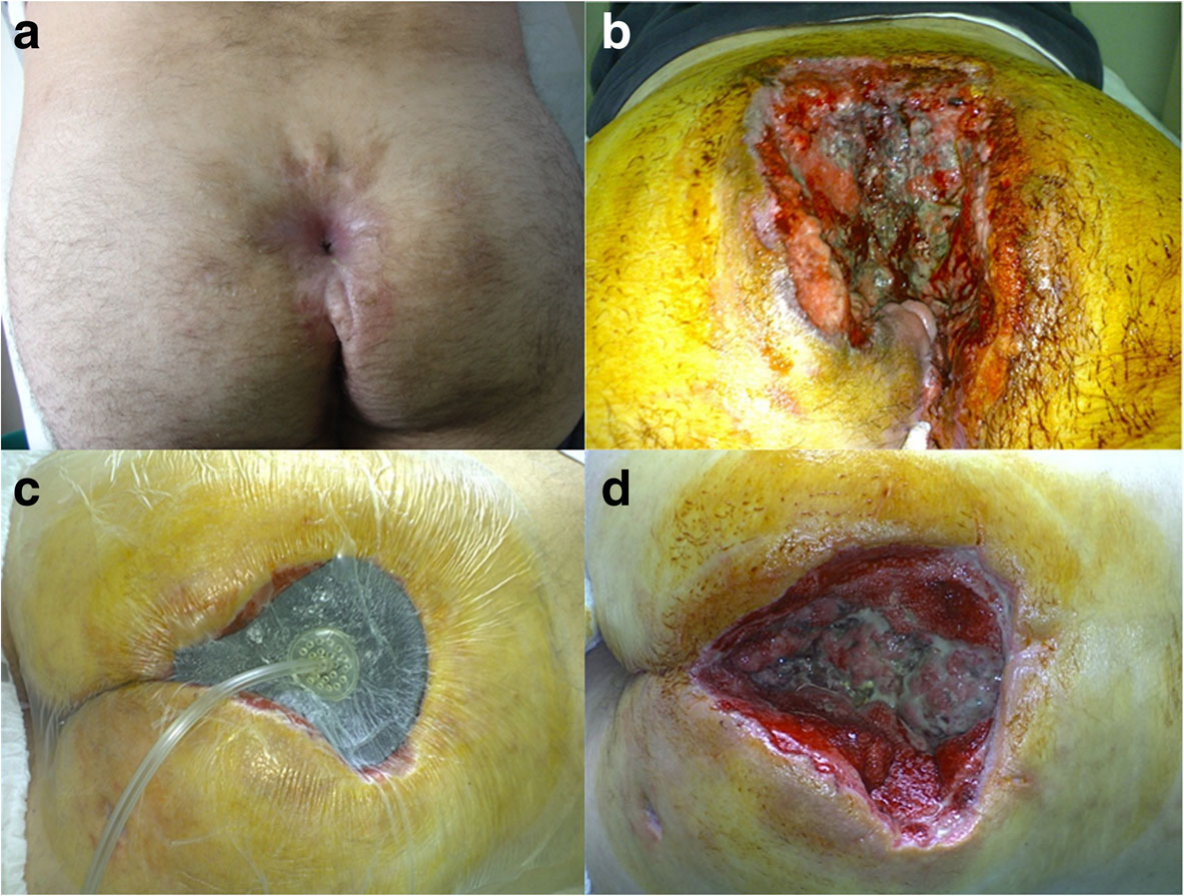
**a** Incomplete healing due to an anal canal fistula. **b** Wide local resection was performed to remove the anal canal fistula. **c** Management of the traumatic area with the vacuum-assisted closure (VAC) system. **d** Squamous cell carcinoma developed over the granulation tissue

A 60-year-old man with chronic and complex sacrococcygeal pilonidal disease (SPD) was referred to our outpatient department for evaluation and management. The SPD had manifested 7 years prior to this admission. The patient reported several episodes of pilonidal abscess formation, which had resolved with either spontaneous drainage and discharge or topical incision. His past medical history was unremarkable. The patient was morbidly obese (body mass index (BMI) 49), but his nutritional status was good. Clinical examination revealed chronic SPD with complex fistulas that had formed through chronic inflammation. He underwent wide surgical excision of the pilonidal sinus, including all fistulas, and histopathological examination of the specimen revealed chronic inflammation. The surgical trauma was left to heal by secondary intention, but even 6 months later, the healing process was poor, with seropurulent discharge detected (Fig. 1a). Subsequently, colonoscopy and a percutaneous fistulography were performed, revealing an anal canal-pilonidal fistula. He underwent a more radical surgical resection (Fig. 1b), with a prophylactic loop colostomy to protect the wound. To facilitate healing, the wound was closed using the vacuum-assisted closure (VAC) technique (V.A.C; KCI International, San Antonio, TX) (Fig. 1c). However, the healing made slow progress. Multiple biopsies were then taken from the surgical site, which revealed the development of SCC (Fig. 1d).

A new colonoscopy was scheduled, and biopsies of the anal canal were obtained. These confirmed the development of SCC. Computed tomography (CT) and magnetic resonance imaging (MRI) revealed SCC partial invasion of the coccyx and sacrum. The inguinal lymph nodes were clinically impalpable, and imaging confirmed that they were normal. We decided that an aggressive surgical approach was warranted and performed abdominoperineal resection, followed by resection of the invaded traumatic area, complete removal of the coccyx, and partial resection of the sacrum. Perforator-based flaps were used to reconstruct the defected area. The localization of perforators around the sacrum was established preoperatively by a handheld Doppler ultrasound scanner. The pedicle was secured; the rest of the flap was raised; and once the flap perfusion was satisfactory, it was carefully lifted from the donor bed and rotated around this pedicle into the recipient defect. The donor defect was closed primarily.

**Fig. 2:**
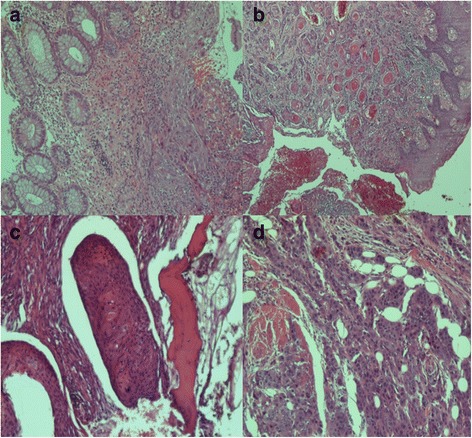
Histopathological findings. **a** Squamous cell carcinoma invading the wall of the large bowel (H&E ×100). **b** Invasion of the dermis by the neoplasm, which shows keratinization in this area (H&E ×40). **c** Area of bone invasion by the neoplasm (H&E ×100). **d** Poorly differentiated area of the neoplasm invading muscle (H&E ×100)

Histological examination revealed moderately to poorly differentiated SCC. The cells were medium to large, with moderately pleomorphic nuclei. There was focal keratinization, and the tumor exhibited a predominantly solid and focally trabecular pattern of growth. The neoplastic cells infiltrated the large bowel wall, dermis, bone, and skeletal muscle (Fig. 2a–d).

The patient was treated with adjuvant radiation therapy postoperatively. No adjuvant chemotherapy was offered. Despite the aggressive surgical intervention, the prognosis was poor. Recurrence was found in the sacrum and para-aorta lymph nodes 9 months after the radical surgery, and the patient died of a respiratory infection soon after this.

## Discussion

SPD frequently occurs as a chronic skin infection in the region of the buttock crease or natal cleft [1, 10]. It is assumed that the disease results from the in-growth of a hair from the surrounding skin [11]. Karydakis proposed three main factors to explain how these hairs make their way into the tissues: the *invader*, which is the loose hair; the *force*, which causes the in-growth; and the *vulnerability* of the skin to the in-growth of the hair at the depth of the natal cleft [12]. A single midline sinus posterior to the anus is characteristic, and while most affected patients present with acute abscess, some suffer chronic infection with discomfort and a purulent discharge [13]. Additional sinuses are also frequent, often with lateral openings [14]. Pilonidal sinuses often penetrate deep into the ischioanal fossa; however, they would not be expected to penetrate the sphincters and involve the anus [7, 10]. In our patient, the fistula involved the anal canal.

Malignant transformation is a rare but well-known complication of long-standing SPD, occurring in approximately 0.1% of patients with recurrent and complex SPD [2, 8, 9, 15, 16]. SCC is the most common carcinoma associated with chronic SPD, although basal cell carcinoma, sweat gland adenocarcinoma, and verrucous carcinoma have also been reported [2, 5, 17]. The pathogenesis of malignant transformation in SPD is thought to be similar to that associated with other chronic ulcerative and scarifying cutaneous disorders, such as Marjolin’s ulcer [2]. It is believed that this process, on the molecular basis, is caused by the release of free oxygen radicals by activated inflammatory cells, inducing genetic damage and neoplastic transformation [9]. The normal repair DNA (deoxyribonucleic acid) mechanism is also impaired in chronic inflammation, which predisposes to malignancy [9, 18]. These predisposing factors result in congenital duplications of the anorectal mucosa, focal adenomatous hyperplasia of the anal glands, and cancerous transformation of rectal mucosal cells that have migrated into the fistulae [19]. Immunosuppression and human papilloma virus infection may also be predisposing factors that induce and accelerate the transformation [20, 21].

Pilonidal carcinoma can be diagnosed by gross inspection, revealing an ulcer with a friable, rapidly growing, bleeding, and fungating margin, in the pilonidal cyst or sinus [22]. Multiple biopsies of the margin of the ulcer provide the histological diagnosis [15, 16]. Occasionally, the lesion is discovered incidentally in an otherwise routine histological examination of a specimen [22]. In the present case, carcinoma was suspected when the surgical wound would not heal, but the initial histopathogical examination did not reveal evidence of malignancy. It must be emphasized that all pilonidal disease specimens should be sent routinely for histopathological evaluation to rule out malignancy.

The most important consideration in the differential diagnosis of pilonidal carcinoma is pseudocarcinomatous hyperplasia of the squamous epithelium [15, 22]. In contrast to reactive hyperplasia, carcinoma will show more nuclear pleomorphism, a greater degree of dyskeratosis, atypical mitotic figures, and invasion of the surrounding connective tissue stroma [15]. As cellular atypia and an increased mitotic rate may also be seen in pseudocarcinomatous hyperplasia, adequate sampling tissue for histological examination must be taken to establish the diagnosis [15]. Preoperative evaluation should include rectal and sigmoidoscopic examinations, and the inguinal lymph nodes must be carefully palpated. CT or MRI images are indicated to demonstrate local extent, to detect intra-abdominal metastases, including the spread to the iliac and para-aortic lymph nodes, and to complement the physical examination of the inguinal lymph nodes [9, 16]. Lumbosacral and coccyx spine films should also be taken to exclude osteomyelitis or bone invasion by the tumor [15].

When there is no evidence of inguinal adenopathy, wide surgical excision is indicated, using strict oncologic techniques of en bloc resection and minimizing violation of the tumor margins and the lesion [21]. Presacral fascia, gluteal muscle, a wide margin of skin and subcutaneous tissue, and, if required, the sacrum and coccyx, should be excised [16]. Prophylactic inguinal node dissection is not recommended [15]. Surgery for superficial SCC has good results [5]. Complete excision by conventional surgery is not feasible when the tumor has become adherent to the sacrum and/or coccyx and has perforated the sacral fascia [4]. Local recurrence after surgery is common [4, 9]. Recurrence rates reach 50% and usually appear 9–16 months after surgery [4]. Adjuvant radiotherapy within a generous margin can decrease local recurrence to 30% [9]. Conversely, the role of adjuvant chemotherapy remains unclear, although it may be effective in combination with resection and radiotherapy for high-risk lesions [4–6]. Re-excision of a local recurrence may prolong survival [9]. The 5-year survival rate of patients with SPD complicated by SCC is almost 55–61% [16, 21]. It was reported that there is no relationship between the degree of tumor differentiation and survival [4].

## Conclusions

In conclusion, the development of SCC in chronic pilonidal disease is a rare but serious complication. Symptoms are usually attributed to the SPD, and diagnosis is often made late by histological examination of biopsies. Malignant transformation should be suspected in chronic SPD with recurrent episodes of inflammation, repeated purulent discharge, poor healing, and chronic complex fistulas. All specimens of pilonidal disease should be sent routinely for histopathological evaluation. Histopathological studies of multiple biopsies allow us to make an accurate diagnosis of SCC and initiate aggressive surgical excision, with or without adjuvant radiotherapy, as the appropriate treatment.

## Abbreviations

BMI: Body mass index
CT: Computed tomography
DNA: Deoxyribonucleic acid
MRI: Magnetic resonance imaging
SCC: Squamous cell carcinoma
SPD: Sacrococcygeal pilonidal disease
VAC: Vacuum-assisted closure
